# Epidemiology of SARS-CoV-2 in Kakuma Refugee Camp Complex, Kenya, 2020–2021^1^

**DOI:** 10.3201/eid3005.231042

**Published:** 2024-05

**Authors:** Maurice Ope, Raymond Musyoka, John Kiogora, Jesse Wambugu, Elizabeth Hunsperger, Gideon O. Emukule, Peninah Munyua, Bonaventure Juma, Elizabeth Simiyu, Levan Gagnidze, John Burton, Rachel B. Eidex

**Affiliations:** Centers for Disease Control and Prevention, Nairobi, Kenya (M. Ope, R. Musyoka, E. Hunsperger, G.O. Emukule, P. Munyua, B. Juma, R.B. Eidex);; International Rescue Committee, Nairobi (J. Kiogora);; United Nations High Commissioner for Refugees, Kakuma, Kenya (J. Wambugu);; International Rescue Committee, Kakuma (E. Simiyu);; International Organization for Migration, Nairobi (L. Gagnidze);; United Nations High Commissioner for Refugees, Nairobi (J. Burton)

**Keywords:** COVID-19, SARS-CoV-2, severe acute respiratory syndrome coronavirus 2, viruses, respiratory infections, zoonoses, refugees, epidemiology, Kenya

## Abstract

Understanding SARS-CoV-2 infection in populations at increased risk for poor health is critical to reducing disease. We describe the epidemiology of SARS-CoV-2 infection in Kakuma Refugee Camp Complex, Kenya. We performed descriptive analyses of SARS-CoV-2 infection in the camp and surrounding community during March 16, 2020‒December 31, 2021. We identified cases in accordance with national guidelines.We estimated fatality ratios and attack rates over time using locally weighted scatterplot smoothing for refugees, host community members, and national population. Of the 18,864 SARS-CoV-2 tests performed, 1,024 were positive, collected from 664 refugees and 360 host community members. Attack rates were 325.0/100,000 population (CFR 2.9%) for refugees,150.2/100,000 population (CFR 1.11%) for community, and 628.8/100,000 population (CFR 1.83%) nationwide. During 2020–2021, refugees experienced a lower attack rate but higher CFR than the national population, underscoring the need to prioritize SARS-CoV-2 mitigation measures, including vaccination.

In late 2019, a cluster of pneumonia cases caused by a novel coronavirus later named SARS-CoV-2 was identified in Wuhan, China (*1*). The virus spread rapidly in China and globally; the World Health Organization (WHO) declared a pandemic on March 11, 2020 (*2*). The disease caused by SARS-CoV-2 was designated by WHO as COVID-19. Some COVID-19 patients develop severe disease requiring hospitalization in intensive care unit; the risk for severe disease was associated with increasing age, socioeconomic status and underlying conditions (*3*). To contain the pandemic, many countries restricted international travel, implemented social distancing, and encouraged the use of face masks and frequent handwashing and sanitizing (*1*).

The United Nations High Commissioner for Refugees (UNHCR) estimates that as of December 2020, there were 20 million refugees and 4.1 million asylum seekers globally, most of whom were living in low- and middle-income countries, usually with weak health systems and poor capacity to manage severe COVID-19 (*4*). As of December 2021, Kenya hosted 540,068 refugees in urban and camp settings (*5*). The number of refugees into Kenya increased steadily during 2019‒2021 (*5*). During the COVID-19 pandemic, the Kakuma Refugee Camp Complex (KRCC) was home to ≈40% of refugees in Kenya. To prevent the introduction and spread of the virus in refugee camps, UNHCR-Kenya and the government of Kenya implemented mitigation measures, including restriction of movement into and out of the camps.

Refugees are at an increased risk for poor health outcomes (*4*) for several reasons: difficulties in access to linguistically and culturally appropriate health related information (*6*); inadequate nutrition, partly because dependence on food rations (*7*); and poor access to adequate health services, including testing for SARS-CoV-2 and management of COVID-19. They often live in remote locations in crowded shelters with poor access to water, sanitation, and hygiene facilities, thus making implementation and enforcement of COVID-19 mitigation measures such as social distancing, handwashing, isolation, and quarantine challenging (*2*). Moreover, some essential services offered to refugees, such as food distribution and refugee registration, repatriation, and resettlement, cause refugees to congregate, increasing the likelihood of SARS-CoV-2 transmission. In addition, humanitarian workers providing services to refugees may travel from urban areas with a high prevalence of SARS-CoV-2 to the refugee camps and may transmit disease into refugee settings.

The epidemiology of SARS-CoV-2 infection in refugee camps is not well described. Given the unique setting and vulnerabilities of this population, it may also be different from the host community, national, or even global epidemiology. Understanding the epidemiology of SARS-CoV-2 infection in refugee populations may lead to targeted interventions and messaging, which can be different from the messaging and interventions designed for the general population. We sought to describe the epidemiology of SARS-CoV-2 infection in the KRCC during the pandemic and compare it with the national situation.

Kenya Ministry of Health reviewed this project and determined that it was a nonresearch programmatic activity to inform public health prevention strategies and did not require local Institutional Review Board approval. This activity was reviewed by CDC and was conducted consistent with applicable federal law and CDC policy (e.g., 45 C.F.R. part 46.102(l)(2), 21 C.F.R. part 56; 42 U.S.C. §241(d); 5 U.S.C. §552a; 44 U.S.C. §3501 et seq.).

## Methods

### Study Site and Population

Turkana West subcounty is located in Turkana County in northwestern Kenya. It shares international boundaries with Uganda to the west and South Sudan to the northwest and is ≈600 km from Nairobi (*8*). It hosts Kakuma refugee camp and Kalobeyei integrated settlement, which comprise the current KRCC. Kakuma refugee camp was established in 1992 as a single camp; it is divided into 4 sections (Kakuma 1, 2, 3 and 4). In 2016, the population of Kakuma refugee camp had exceeded its maximum capacity, prompting the establishment of Kalobeyei integrated settlement to provide additional accommodation for refugees. In this new settlement, located 25 km away from Kakuma refugee camp, refugees are integrated into host communities (*9*). The host community mainly consists of pastoralists who live throughout Turkana West subcounty; they are able to mix with refugees and can access health services within the refugee camps (*9*) (Appendix Figure, https://wwwnc.cdc.gov/EID/article/30/5/23-1042-App1.pdf).

During March 1, 2020‒December 31, 2021, KRCC hosted 196,050‒219,901 refugees and asylum seekers, most of whom were from South Sudan, Democratic Republic of the Congo, Burundi, Ethiopia, and Sudan (*5*). For this analysis, we used the population at the midpoint in time, February 2021, as our denominator; it was 204,309 (*5*). At the same time, an estimated 239,627 host community members were living in Turkana West subcounty. The main UNHCR healthcare implementing partner is the International Rescue Committee (IRC).

### Testing for SARS-CoV-2

Since March 11, 2020, when WHO declared COVID-19 a pandemic, patients meeting the definition of a suspected case of COVID-19 and their close contacts were prioritized for testing for SARS-CoV-2 (*10*,*11*). Specimens for SARS-CoV-2 testing were collected from the patient’s upper and lower respiratory system in accordance with guidelines from Kenya Ministry of Health (*10*). In May 2020, as we learned that presymptomatic persons could spread the virus (*12*), UNHCR recommended expanded testing to include refugees and humanitarian workers each time they traveled in or out of the camp. Beginning in April 2021, testing was required for refugees on voluntary repatriation to their country of origin and for those being prepared for any surgical procedure. Of note, the case definition of COVID-19 changed over time; fever and history of travel were required initially, and later, persons with other symptoms of COVID-19 but no history of travel met the definition of a suspected case and were eligible for testing. Those changes were consistent with WHO guidelines and were officially communicated by the Kenya Ministry of Health through official notifications and published on the Ministry of Health website (*10*,*11*). SARS-CoV-2 infections were identified following national guidelines and a confirmed SARS-CoV-2 case was anyone with laboratory confirmation of SARS-CoV-2 infection either by real-time reverse transcription PCR (real time RT-PCR), GeneXpert SARS-CoV-2 rapid real-time RT-PCR (GeneXpert; Cephid, https://www.cepheid.com), or antigen rapid diagnostic testing (Ag RDT), irrespective of clinical signs or symptoms (*11*). A COVID-19 case-patient was any symptomatic person who tested positive for SARS-CoV-2.

**Figure 1:**
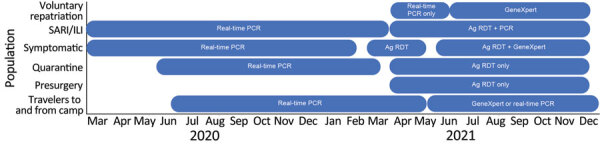
Testing algorithm and timelines for priority groups in Kakuma Refugee Camp Complex, Kenya, March 2020‒December 31, 2021. Voluntary repatriation was halted in 2020 because of the pandemic and resumed in April 2021. Symptomatic persons were initially tested for SARS-CoV-2 using real-time PCR; starting April 2021, they were tested used only Ag RDT; starting in June 2021, positive samples were confirmed using GeneXpert (Cephid, https://www.cepheid.com). Persons with SARI/ILI were initially tested using real-time PCR; starting April 2021, they were tested using both Ag RDT and real-time PCR. Ag RDT, antigen rapid diagnostic testing; ILI, influenza-like illness; SARI, severe acute respiratory illness.

Severe acute respiratory illness (SARI) and influenza-like illness (ILI) surveillance was established in Kakuma refugee camp in 2007. Samples from persons meeting the SARI/ILI case definitions were tested for influenza viruses, respiratory syncytial virus, human metapneumovirus, and parainfluenza virus. The case definitions for SARI and ILI are described elsewhere (*13*). Effective March 16, 2020, specimens were also tested prospectively for SARS-CoV-2 using real-time RT-PCR at the US Centers for Disease Control and Prevention (CDC)–supported Kenya Medical Research Institute (KEMRI) laboratory in Nairobi. Over time, the testing algorithm for SARS-CoV-2 was modified as the testing capacity was expanded from centralized real-time RT-PCR testing in Nairobi to additional laboratories performing real-time RT-PCR, GeneXpert, and Ag RDT testing (Figure 1). In KRCC, Ag RDT testing was available in April 2021 and GeneXpert testing in June 2021. To ensure timely isolation of COVID-19 cases, SARI and ILI cases in which there was a strong clinical suspicion of COVID-19 were tested using Ag RDT at the IRC laboratory. Persons experiencing symptoms, under quarantine, or being prepared for surgery were also tested using Ag RDT. During April‒June 2021, the International Organization for Migration (IOM) laboratory tested refugees on resettlement and humanitarian workers using real time RT-PCR; thereafter, either GeneXpert or real time RT-PCR was used. AMPATH tested specimens from Turkana West Subcounty beginning in May 2021 and Lancet laboratories beginning in June 2021, using real-time RT-PCR whenever it was not feasible to test the specimens either at IRC or CDC-supported KEMRI laboratories.

### Study Design

We conducted a descriptive analysis of routinely collected data from March 2020‒December 31, 2021, on demographics, SARS-CoV-2 testing results, and clinical outcomes of COVID-19 in KRCC. We used multiple sources of SARS-CoV-2 infection data for Turkana West subcounty and KRCC, each with its own limitations. 

#### National SARS-CoV-2 Testing Repository

On June 16, 2020, the Kenya Ministry of Health established a national repository for SARS-CoV-2 testing at the National Public Health Laboratory (NPHL). All laboratories accredited to perform SARS-CoV-2 testing were required to submit all test results to the national SARS-CoV-2 testing repository through a secure web link. This dataset included all persons tested for SARS-CoV-2 using Ag RDT, GeneXpert, and real-time RT-PCR. For the purposes of our analysis, the national SARS-CoV-2 testing repository data was limited to Turkana West subcounty.

#### National Line List of SARS-CoV-2‒Positive Cases

Upon the detection of the first case of SARS-CoV-2 in Kenya on March 14, 2020, the national Public Health Emergency Operation Center (PHEOC) maintained a line list of all laboratory-confirmed cases. This list was regularly updated for symptomatic cases with outcome of illness.

#### Line List of Positive Cases in the Refugee Camp 

Upon the detection of the first SARS-CoV-2 infection in the camp, UNHCR and IRC maintained a line list of laboratory-confirmed SARS-CoV-2 cases. For symptomatic cases, this list was regularly updated with outcome of illness.

### Data Management

We primarily used data from the national SARS-CoV-2 testing repository, line list of SARS-CoV-2 positive cases maintained by IRC, and the national PHEOC. We restricted the analysis to KRCC and calculated SARS-CoV-2 testing rates, SARS-CoV-2 infection attack rates, and COVID-19 case-fatality ratios (Table).

**Table:** Data sources used for analysis of SARS-CoV-2 infection, Kakuma Refugee Camp Complex, Kenya, 2020–2021*

Data source	Refugees	Host	Kenya
National SARS-CoV-2 testing repository	SARS-CoV-2 testing rate	SARS-CoV-2 testing rate	SARS-CoV-2 testing rate
National line list of positive cases	NA	COVID-19 attack rate and case-fatality ratio	COVID-19 attack rate and case-fatality ratio
UNHCR/IRC line list of positive cases from the refugee camp	COVID-19 attack rate and case-fatality ratio	NA	NA
Source of population size	Population statistics from UNHCR	2019 National Housing and Population Census	2019 National Housing and Population Census

*IRC, International Rescue Committee; NA, not applicable; UNHCR, United Nations High Commissioner for Refugees.

We defined refugees as persons living in KRCC or who were noted as a refugee in the database and host community members as nonrefugees who lived in Turkana West subcounty and were not employed as humanitarian workers. We excluded humanitarian workers from this analysis because it was unclear how many were working in the camp during the pandemic. Moreover, the workers traveled several times in and out of the camp as part of their work, thus requiring testing multiple times, unlike refugees and host community members, who moved in and out of the camps much less frequently.

We performed statistical analysis using SAS software version 9.4 (SAS Institute Inc., https://www.sas.com) and plotted graphs using R version 4.1.1 (The R Foundation for Statistical Computing, https://www.r-project.org). We estimated attack rates with their associated 95% CIs for refugees and the host community using population statistics from UNHCR (*14*) and the 2019 national population census (*15*). We estimated attack and testing rates over time using locally weighted scatterplot smoothing, a localized weighted regression with a span of 20% of the nearest point and heavier weighting of closer points. To avoid negative attack rates due to the effect of smoothing caused by values closer to zero, we fitted them on a log scale and then applied exponentiation to get results on the original scale.

## Results

The first positive SARS-CoV-2 case from KRCC was detected on May 22, 2020, in a refugee camp 2 months after the first case of SARS-CoV-2 was reported in Kenya. This case-patient was tested because he had traveled from Nairobi to the camp. By December 31, 2021, a total of 1,024 SARS-CoV-2 infection cases were reported in KRCC; among those, 664 (63.4%) were refugees and 360 (36.6%) members of the host community. The median patient age was 25 (1–102) years; 498 (51.0%) patients were male. Nationally, a total of 297,155 SARS-CoV-2 cases were reported as of December 31, 2021.

During March 2020‒December 31, 2021, a total of 23 COVID-19 deaths were reported in KRCC and 5,442 nationally. The overall CFRs were 2.25% (95% CI 1.29%–3.20%) for KRCC and 1.83% (95% CI 1.78%–1.87%) for Kenya. Of the 23 deaths in KRCC, 19 deaths (CFR 2.86%, 95% CI 1.52%–4.20%) were reported among refugees, and 4 deaths (CFR 1.11%, 95% CI 0.00%–2.33%) among the host community.

**Figure 2:**
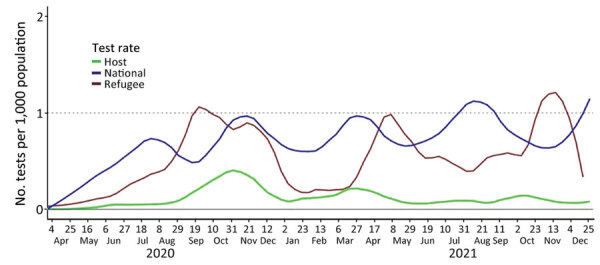
Weekly COVID-19 SARS-CoV-2 testing rates per 1,000 population for the general population and among host and refugee populations in Kakuma Refugee Camp Complex, Kenya, March 16, 2020‒December 31, 2021.

During the same period, a total of 18,864 SARS-CoV-2 tests from Turkana West subcounty were performed. Weekly variation in the testing rates during the pandemic (Figure 2) resulted in part from challenges in obtaining supplies for specimen collection and testing; hence, it is likely that many SARS-CoV-2 infection cases were missed and not reported. The testing rate for SARS-CoV-2 was lower than the WHO recommended minimum weekly testing rate of 1/1,000 population, among all 3 populations in our analysis: refugees (10,689 tests; 0.52/1,000 population), host community members (2,635 tests; 0.11/1,000 population), and nationally (3,260,483 tests; 0.69/1,000 population).

**Figure 3:**
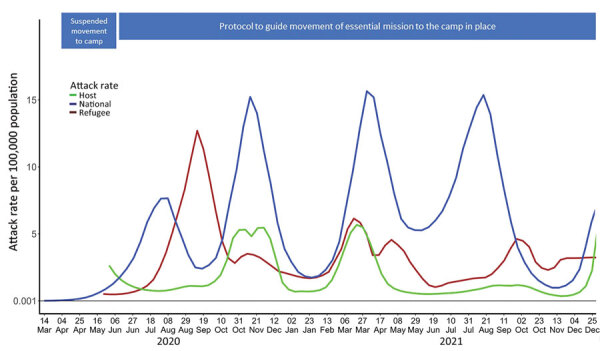
Weekly SARS-CoV-2 infection attack rates per 100,000 population for the general population and among host and refugee populations in Kakuma Refugee Camp Complex, Kenya, March 16, 2020‒December 31, 2021.

The overall SARS-CoV-2 infection attack rates per 100,000 persons was 325.0 (95% CI 300.1–349.9) among refugees , 150.2 (95% CI 134.5–165.9) in the host community, , and 628.8 (95% CI 626.6–631.1) nationally. The COVID-19 pandemic in Kenya occurred in waves. Nationally, there were 5 waves: June‒August 2020, September‒December 2020, February‒May 2021, May‒September 2021, and November‒December 2021 (Figure 3). Similarly, refugee and host communities experienced waves of infections. The highest attack rate among refugees and host community members during the first wave, and the subsequent waves were of a lesser magnitude than the previous waves. The third national wave coincided with an increase in attack rate among refugees and host community. In contrast, during the fourth national wave there was no increase in attack rate among refugees and host community members.

## Discussion

This analysis found that refugees were severely negatively affected by COVID-19 during the pandemic. Refugees experienced a lower attack rate but a higher CFR than the population of Kenya as a whole. The low attack rate among refugees compared with national rates is similar to findings in Jordan’s refugee camps (*16*), which may suggest that refugees were not responsible for the continued spread of SARS-CoV-2 but instead were affected by external SARS-CoV-2 transmission. We noted that the pandemic occurred when the Kenya government had given a nonnegotiable ultimatum for closure of refugee camps that was not enforced at the time (*17*); stigmatization, increased restriction, or crackdowns on refugees were possible if they were thought to be responsible for driving infection. At the beginning of the pandemic there were fears of catastrophic effects of COVID-19 among refugee settings because those populations were already vulnerable (*18*). We found a higher CFR among refugees compared to national rates. The low testing rate among host community members may partly explain a lower attack rate, whereas the lower CFR observed among host community members could be a consequence of poor documentation of deaths among this population in Turkana West subcounty. 

We cannot fully explain why the refugees experienced a higher CFR than the host and national populations. One possible reason is that, unlike in the host and Kenya populations, all deaths among refugees were meticulously investigated to determine if the cause of death was COVID-19. This process included collection and testing of nasopharyngeal swabs from deceased refugees at both community and health facility levels, potentially inflating the CFR. In addition, contributing factors such as malnutrition, other diseases, or poor health status could put refugees at higher risk for severe disease and death. Unfortunately, that information was not available for analysis.

Measures put in place to either delay introduction of the virus into the camps or contain transmission within Kenya had positive effects and some unintended consequences. In April 2020, the government of Kenya imposed restriction on movement of persons from SARS-CoV-2‒infected areas and a lockdown in urban areas (*19*,*20*). Similarly, UNHCR restricted movement into and out of the camps to delay introducing the virus in the refugee camps to enhance pandemic control. Whereas the national lockdown and restriction of movement from SARS-CoV-2‒infected areas were imposed with good intentions, they may have had unintended consequences on refugees. Refugees who were living on daily wages in urban settings experienced job losses that caused them to migrate from urban settings with high SARS-CoV-2 attack rates to the camps, introducing SARS-CoV-2 into the camps, as happened with the first case in KRCC.

After the introduction of the virus to the camp, the first wave among refugees had very high attack rates at a time when the testing rates were very low. The high attack rates were partly caused by inability to significantly reduce contact with many undiagnosed case-patients living with other refugees in crowded shelters. At that time, the estimated population of refugees in the camp was 196,120 in an expected capacity of <100,000 (*5*). Also, because most of the cases reported were asymptomatic at that time, there was inadequate information among refugees on SARS-CoV-2. Poor adherence to COVID-19 mitigation measures among refugees was not uncommon, which may partly explain the high attack rates reported. In contrast, the attack rate among host community members was low during the first wave, possibly because it was a sparsely populated rural population, which reduced the risk for transmission.

Although we did not test for the SARS-CoV-2 variants circulating in the refugee camps, it is likely that that the variants that were predominant nationally were also circulating in the refugee camp. The third national wave commenced in January 2021 when the more transmissible Alpha variant B.1.1.7 was detected in Kenya (*21*); during that time, there was a similar increase in attack rate among refugees and host community members. The Delta variant B.1.617.2 was detected in Kenya in March 2021, co-circulating with the Alpha variant (*22*). The Delta variant took over as the predominant variant during the fourth national wave. It is unclear why, during the period when the Delta variant was predominant in Kenya, the attack rate increased nationally but not among refugees.

We also found that testing was low for refugees nearly throughout the pandemic; some SARS-CoV-2 infections were undetected and thus the reported attack rates are likely an underestimate. The introduction of Ag RDTs and GeneXpert testing in the camp led to improvement in testing rate of refugees, although it did not reach the minimum test per population rate recommended by WHO. The improved testing capacity came late in the pandemic, when it was no longer tenable to perform contact tracing for all confirmed cases. The low testing rate among refugees in KRCC is consistent with findings among other refugee populations (*23*). 

The first limitation of this descriptive analysis is that we relied on routine data collected by different entities which had gaps, and hence, we had to cross-check with other datasets to have a complete dataset for analysis. To do that, we had to make assumptions because of the unique deficiencies in each dataset: the national SARS-CoV-2 testing repository was established after SARS-CoV-2 cases were detected in the camp; the repository was not routinely checked for errors; multiple tests for the same person were captured even if they were done almost at the same time; and manual transfer of data using Microsoft Excel spreadsheets from the testing laboratory to the national SARS-CoV-2 repository could have led to some errors. In addition, we were unable to identify persons who had multiple tests performed during the same infection or those with prolonged shedding of the virus. Prolonged detection of viral RNA does not indicate infectiousness (*24*). In our analysis, we reported those infections as separate, thus overestimating the attack rates for refugees and for host and national populations. Those deficiencies likely led to reporting bias. Second, the reported positive cases were based on multiple testing platforms such as RT-PCR, GeneXpert, and Ag RDT, which had different sensitivities and specificities. Ag RDT, for example, had a low sensitivity, potentially resulting in false negatives and resulting in an underestimate of the reported attack rates. Third, since we are comparing attack rates, it would have been better if we adjusted for the varying testing rates among the populations; however, this adjustment was not possible because host and refugee populations were a subset of the national database.

In conclusion, KRCC did not escape the COVID-19 pandemic. Refugees experienced a reported lower attack rate but a higher CFR compared with the national rates. It is unclear if the high CFR among refugees was a result of poor utilization of health services, poor access to healthcare by severe cases, low testing rate, or a general predisposition to ill health because of malnutrition or co-infection with other pathogens. 

Testing is an essential component in controlling the pandemic to ensure early isolation, contact tracing, and case management. To provide evidence on the need for behavior change to mitigate the spread of SARS-CoV-2, a robust testing system is critical. Therefore, to prepare for the next pandemic, it is essential to strengthen laboratory capacity that would serve not only refugees but also rural communities hosting the refugees.

The challenges with reliable SARS-CoV-2 data from this refugee population also emphasizes the need for improved integration of migrant populations into national data systems to better understand differences in disease transmission during outbreaks and pandemics. Our findings underscore the need to prioritize COVID-19 mitigation measures, including vaccination for refugees.

Appendix Additional information about SARS-CoV-2 infections in Kakuma Refugee Camp Complex, Kenya, 2020–2021.
