# Supplementary material for: Epidemiology of SARS-CoV-2 in Kakuma Refugee Camp Complex, Kenya, 2020–2021
Source: Emerg Infect Dis. 2024 May;30(5):900–7. doi: 10.3201/eid3005.231042 (PMC11060438; doi:10.3201/eid3005.231042)
Supplement: Appendix — Additional information about SARS-CoV-2 infections in Kakuma Refugee Camp Complex, Kenya, 2020–2021. [file 23-1042-Techapp-s1.pdf]

Article DOI: http://doi.org/10.3201/eid3005.231042

Epidemiology of SARS-CoV-2 in Kakuma Refugee Camp Complex, Kenya, 2020–2021

Appendix

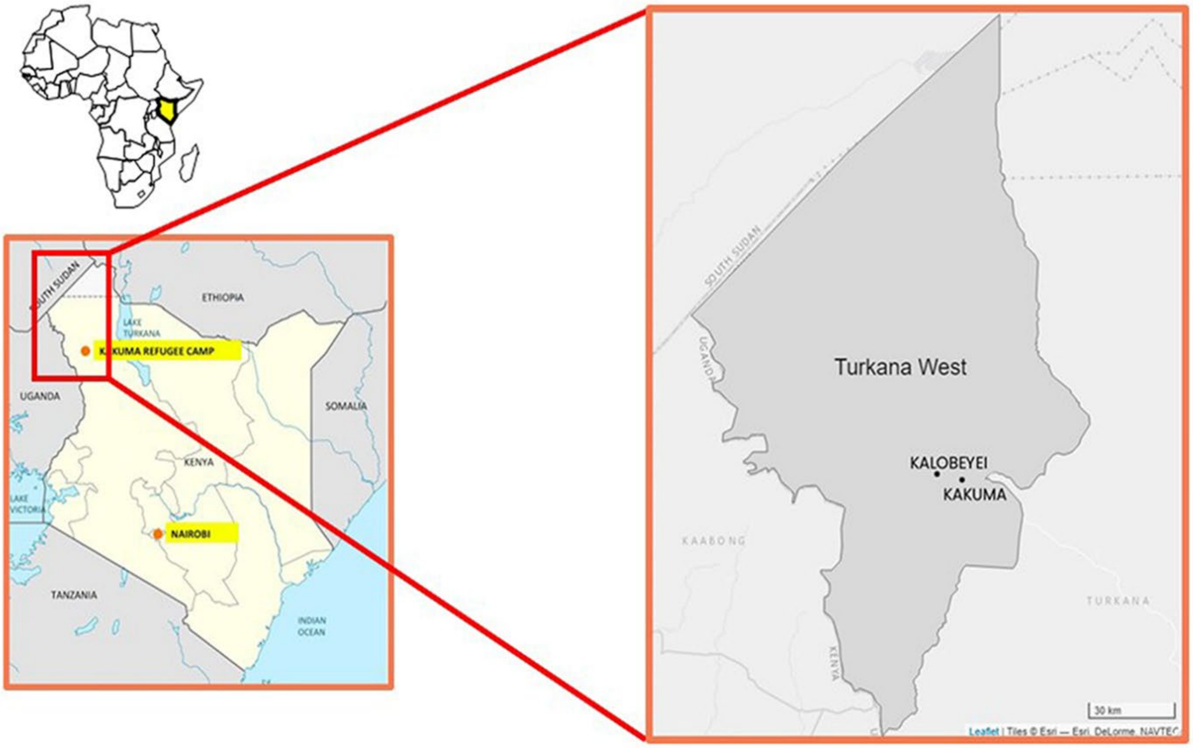

Appendix Figure. Location of Kakuma Refugee Camp Complex within Turkana West subcounty, Kenya.
